# Mental Health Outcomes Among Patients Living in US Counties Lacking Broadband Access and Psychiatrists

**DOI:** 10.1001/jamanetworkopen.2023.33781

**Published:** 2023-09-14

**Authors:** Tarun Ramesh, Ryan K. McBain, Jonathan H. Cantor, Bradley D. Stein, Hao Yu

**Affiliations:** 1 Department of Population Medicine, Harvard Medical School and Harvard Pilgrim Health Care Institute, Boston, Massachusetts; 2 RAND Corporation, Santa Monica, California; 3 RAND Corporation, Pittsburgh, Pennsylvania

## Abstract

This cross-sectional study identifies the prevalence of counties without psychiatrists and broadband coverage, describes their sociodemographic characteristics, and quantifies their mental health outcomes.

## Introduction

The US has a severe shortage of psychiatrists,^1^ causing individuals with mental health disorders to turn to emergency departments.^2^ Over the past decade, the federal government has emphasized a multipronged strategy to bolster the psychiatric workforce, including increasing psychiatry residency slots and incentivizing clinicians to practice in underserved areas.^3^

With the onset of COVID-19, telehealth provided a vehicle for expanding psychiatric care access. However, in 2019, 21.3 million US residents, including nearly a quarter of rural residents, still lacked broadband access, forcing them to use only telephone consultation or no telehealth services altogether.^4^ This analysis identifies the prevalence of counties without psychiatrists and broadband coverage, describes their sociodemographic characteristics, and quantifies their mental health outcomes.

## Methods

This cross-sectional study was deemed exempt by the Harvard Pilgrim Health Care Institute institutional review board, and informed consent was not required because we used publicly available data. We followed the STROBE reporting guideline.

We used county-level information, including sociodemographic characteristics from the American Community Survey 5-year estimates, psychiatrist workforce from the Health Resources and Services Administration (HRSA) Area Health Resources Files, and the Federal Communications Commission’s characterization of insufficient broadband (Table).^5^ County-level measures of mental health outcomes included rates of adult depression and frequent mental distress, and drug overdose deaths and completed suicides per 100 000 population. The measures were derived by County Health Rankings, which used the US Centers for Disease Control and Prevention (CDC) Behavioral Risk Factor Surveillance Survey to estimate the former 2 measures and the CDC National Vital Statistics System to estimate the latter 2 measures.^6^

**Table.** Logistic Regression Analysis of Characteristics of Counties Without a Psychiatrist and Broadband Coverage^a^

	Adjusted OR (95% CI)	*P* value
Median household income (per $1000)	1.00 (0.99-1.02)	.84
% Unemployed	1.12 (1.02-1.24)	.02
Race and ethnicity		
% Hispanic	0.98 (0.97-0.99)	<.001
% Non-Hispanic Black	0.99 (0.981-1.00)	.12
% Non-Hispanic Other^b^	0.99 (0.97-1.00)	.12
% Non-Hispanic White	1 [Reference]	NA
% Bachelor’s degree	0.92 (0.90-0.94)	<.001
% Uninsured	1.03 (1.00-1.06)	.05
MHPSA	0.83 (0.65-1.06)	.14
Residence		
Rural	3.05 (2.41-3.85)	<.001
Urban	1 [Reference]	NA

Abbreviations: MHPSA, mental health professional shortage areas (designated by the Health Resources and Services Administration); NA, not applicable; OR, odds ratio.

^a^ Rural counties were defined as the 2013 USDA rural-urban continuum codes of 8 or 9. The multivariate model included state fixed effects and the following county-level covariates: median household income (in $1000); percentage unemployed among people 16 years of age and older; percentage Hispanic, percentage non-Hispanic Black, and percentage non-Hispanic other using non-Hispanic White as the reference group; percentage population older than 25 years with a bachelor’s degree; percentage uninsured; MHPSA; rural with urban as the reference. Broadband coverage was determined using the Federal Communications Commission’s definition of high-speed, or broadband internet access, as minimum 25 megabits per second (Mbps) download and 3 Mbps upload. Counties without either or both speed levels were marked as inadequate broadband coverage.

^b^ Race and ethnicity was self-reported in the American Community Survey 5-year estimates, and non-Hispanic other refers to individuals who are Asian, Indigenous, multiracial, or marked other on the American Community Survey.

We identified counties without psychiatrists and broadband coverage in 2020 and assessed their sociodemographic characteristics (Table) through a multivariate logistic regression with state fixed effects. To compare mental health outcomes between counties without psychiatrists and broadband and other counties, we conducted univariate analyses using χ^2^ tests and linear multivariate regressions, controlling for county-level sociodemographic characteristics (Figure). Two-sided *P* < .05 was considered statistically significant. Statistical analysis was conducted using Stata version 18.0 (StataCorp) from December 2022 to May 2023.

**Figure.**
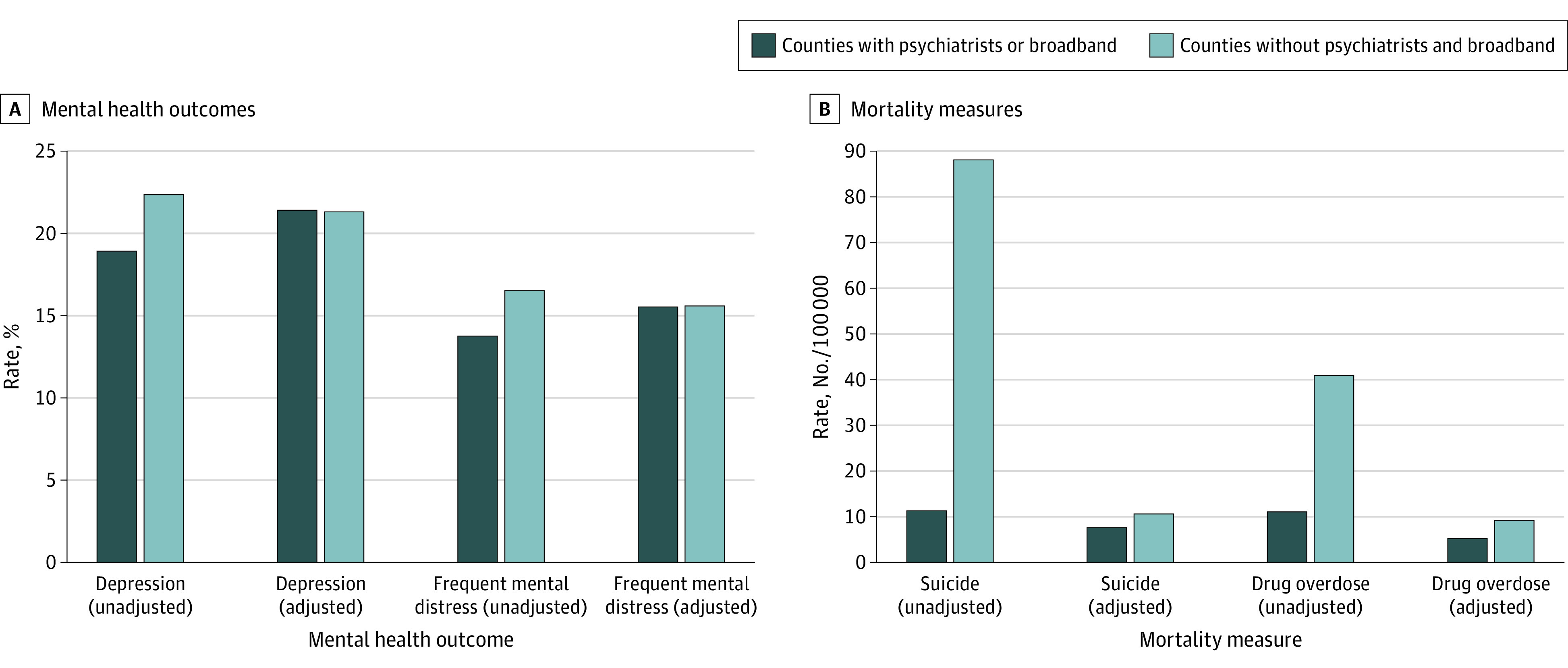
Differences in Survey-Based Mental Health Outcomes and Mortality Measures Between Counties Without Psychiatrists and Broadband and Other Counties A, Depression was defined as an affirmative response to the question, “Have you ever been told you have a depressive disorder (including depression, major depression, dysthymia, or minor depression)?” Frequent mental distress was defined as a response of at least 14 days to the question, “Now thinking about your mental health, which includes stress, depression, and problems with emotions, for how many days during the past 30 days was your mental health not good?” Both measures were estimated by County Health Rankings, which used information from the US Centers for Disease Control and Prevention Behavioral Risk Factor Surveillance Survey. The adjusted percentages were generated through multivariate linear regressions, which included the following county-level covariates: median household income (in $1000), percentage unemployed among people 16 years of age and older; percentage Hispanic, percentage non-Hispanic Black, and percentage non-Hispanic other using non-Hispanic White as the reference group; percentage population older than 25 years of age with a bachelor’s degree; percentage uninsured; mental health professional shortage area; rural with urban as the reference. B, Drug overdose deaths per 100 000 population and completed suicides per 100 000 population in a county were estimated by County Health Rankings, which used information from the US Centers for Disease Control and Prevention National Vital Statistics System. The adjusted percentages were generated through multivariate linear regressions, which included the following county-level covariates: median household income (in $1000); percentage unemployed among people 16 years of age and older; percentage Hispanic, percentage non-Hispanic Black, and percentage non-Hispanic other using non-Hispanic White as the reference group; percentage population older than 25 years of age with a bachelor’s degree; percentage uninsured; mental health professional shortage area; rural with urban as the reference.

## Results

We identified 596 counties (19.0% of all US counties) without psychiatrists and broadband, representing 10.5 million residents. Regression results indicated that these counties were more likely to be rural (adjusted odds ratio [aOR], 3.05 [95% CI, 2.41-3.84]), have higher unemployment rates (aOR, 1.12 [95% CI, 1.02-1.24]), uninsurance rates (aOR, 1.03 [95% CI, 1.00-1.06]), and lower proportions of population with bachelor’s degrees (aOR, 0.92 [95% CI, 0.90-0.94]) and Hispanic individuals (aOR, 0.98 [95% CI, 0.97-0.99]), but were not associated with the HRSA mental health professional shortage area (MHPSA) designation (Table).

The χ^2^ tests found that counties without psychiatrists and broadband had higher rates of adult depression (0.22 [95% CI, 0.13-0.33] vs 0.19 [95% CI, 0.12-0.29]; *P* < .001), frequent mental distress (0.17 [95% CI, 0.09-0.27] vs 0.14 [95% CI, 0.08-0.23]; *P* < .001), drug overdose mortality (40.9 [95% CI, 29.4-55.6] vs 11.1 [95% CI, 5.5-19.7] per 100 000; *P* < .001) and completed suicide (88.1 [95% CI, 70.6-108.4] vs 11.3 [95% CI, 5.5-19.7] per 100 000; *P* < .001) compared with other counties. The multivariate regressions indicated that the adjusted difference between the 2 county groups remained statistically significant for drug overdose mortality (9.2 [95% CI, 8.0-10.5] vs 5.2 [95% CI, 4.9-5.6] per 100 000; *P* < .001) and completed suicide (10.6 [95% CI, 8.9-12.3] vs 7.6 [95% CI, 7.0-8.2] per 100 000; *P* < .001) while the adjusted difference was not significant for the other 2 measures (Figure).

## Discussion

We found more than 10 million residents of counties in 2020 without psychiatrists and broadband coverage, primarily rural counties and counties with lower socioeconomic status, along with worse mental health outcomes. Our finding suggests that lacking access to virtual and in-person psychiatric care continues to be a key factor associated with adverse outcomes after controlling for county-level sociodemographic status. Future research should examine whether recent legislation, including the Consolidated Appropriations Act of 2021 and American Rescue Plan, which expanded psychiatry residency slots and broadband infrastructure, reduces these dynamics.

Importantly, MHPSA designation was not associated with whether a county had no psychiatrists and broadband. The designation brings additional funding to recruit clinicians to underserved areas. However, it does not take into account the growing importance and increased availability of telepsychiatry, an important shortfall that will grow as telehealth services are increasingly adopted.

Our study’s limitations include its cross-sectional nature, its examination of county-level associations, its exclusion of nonpsychiatrist mental health clinicians, and the fact that broadband coverage and the presence of psychiatrists are necessary but not sufficient to enhance uptake of services. Federal and state-level investments in broadband and the psychiatric workforce are needed to improve digital equity and psychiatric care access.

## Notes

Supplement.
Data Sharing Statement
