# Supplementary material for: Mental Health Outcomes Among Patients Living in US Counties Lacking Broadband Access and Psychiatrists
Source: JAMA Netw Open. 2023 Sep 14;6(9):e2333781. doi: 10.1001/jamanetworkopen.2023.33781 (PMC10502528; doi:10.1001/jamanetworkopen.2023.33781)
Supplement: Supplement. — Data Sharing Statement [file jamanetwopen-e2333781-s001.pdf]

# Data Sharing Statement

Ramesh. Mental Health Outcomes Among Patients Living in US Counties Lacking Broadband Access and Psychiatrists. *JAMA Netw Open*. Published September 14, 2023. doi:10.1001/jamanetworkopen.2023.33781

**Data**
**Data available:** Yes
**Data types:** Data (not involving human participants)
**How to access data:** The Area Health Resources Files are publicly available (https://data.hrsa.gov/data/download). The American Community Survey five-year population estimates are also publicly available (https://www.census.gov/data/developers/data-sets/acs-5year.html).
**When available:** With publication

**Supporting Documents**
**Document types:** None

**Additional Information**
**Who can access the data:** Anyone requesting the data.
**Types of analyses:** For research purpose only.
**Mechanisms of data availability:** Without investigator support.
